# Immunohistochemical profiling of benign, low malignant potential and low grade serous epithelial ovarian tumors

**DOI:** 10.1186/1471-2407-8-346

**Published:** 2008-11-26

**Authors:** Véronique Ouellet, Tak Hay Ling, Karine Normandin, Jason Madore, Christian Lussier, Véronique Barrès, Dimcho Bachvarov, Claudine Rancourt, Patricia N Tonin, Diane M Provencher, Anne-Marie Mes-Masson

**Affiliations:** 1 Centre de recherche du centre hospitalier de l'Université de Montréal (CHUM)/Institut du cancer de Montréal, Montreal, Canada; 2 Department of Obstetric/Gynecology, Taipei Medical University-Wan Fang Hospital, Taipei, Taiwan; 3 Department of Pathology, Centre hospitalier de l'Université de Montréal (CHUM), Montreal, Canada; 4 Department of Medicine, Université Laval, Quebec, Canada; 5 Centre de recherche de l'Hôtel-Dieu de Québec, Quebec, Canada; 6 Department of Microbiology-Infectiology, Université de Sherbrooke, Sherbrooke, Quebec, Canada; 7 Department of Human Genetics, McGill University, Montreal, Canada; 8 Research Institute of the McGill University Health Centre, Montreal, Canada; 9 Department of Medicine, McGill University, Montreal, Canada; 10 Division of Gynecologic Oncology/Université de Montréal, Montreal, Canada; 11 Department of Medicine, Université de Montréal, Montreal, Canada

## Abstract

**Background:**

Serous epithelial ovarian tumors can be subdivided into benign (BOV), low malignant potential (LMP) or borderline and invasive (TOV) tumors. Although the molecular characteristics of serous BOV, LMP and low grade (LG) TOV tumors has been initiated, definitive immunohistochemical markers to distinguish between these tumor types have not been defined.

**Methods:**

In the present study, we used a tissue array composed of 27 BOVs, 78 LMPs and 23 LG TOVs to evaluate the protein expression of a subset of selected candidates identified in our previous studies (Ape1, Set, Ran, Ccne1 and Trail) or known to be implicated in epithelial ovarian cancer disease (p21, Ccnb1, Ckd1).

**Results:**

Statistically significant difference in protein expression was observed for Ccnb1 when BOV tumors were compared to LMP tumors (p = 0.003). When BOV were compared to LG TOV tumors, Trail was significantly expressed at a higher level in malignant tumors (p = 0.01). Expression of p21 was significantly lower in LG tumors when compared with either BOVs (p = 0.03) or LMPs (p = 0.001). We also observed that expression of p21 was higher in LMP tumors with no (p = 0.02) or non-invasive (p = 0.01) implants compared to the LMP associated with invasive implants.

**Conclusion:**

This study represents an extensive analyse of the benign and highly differentiated ovarian disease from an immunohistochemical perspective.

## Background

Tumors of the ovary represent a large, heterogeneous and complex group of neoplasms. The majority of these tumors are derived from ovarian surface epithelial cells, from epithelial inclusion cysts confined in the stroma or from the epithelium of the fallopian tube [1,2]. Epithelial ovarian tumors present as different histopathology subtypes among which the serous subtype is the most frequent [reviewed in [3,4]].

Serous tumors can be subdivided into benign (BOV), borderline or low malignant potential (LMP) and invasive (TOV) tumors. BOV tumors are characterized by epithelial proliferation without any stratification of cells. LMP tumors are distinguished from their benign counterpart by the complexity of their architecture and the presence of epithelial budding. LMP tumors show a pluristratified proliferation of the epithelium. LMP tumor cells exhibit some nuclear atypia and show a higher mitotic activity when compared to BOV [reviewed in [5-9]].

In contrast to BOV and LMP tumors, TOVs have the capability of invading the ovarian stroma. TOV tumor cells present severe nuclear atypia and show high mitotic index which usually increase with tumor grade. According to the FIGO criteria, EOCs are graded according to degree of tumor differentiation: LMPs (referred to as grade 0, G0) while TOVs are separated in well (grade 1, G1, low grade, LG), moderately (grade 2, G2), and poorly differentiated tumors (grade 3, G3) [10,11]. However, several papers now support a two-tiered classification that separate invasive tumors into low (G1) and high (G2 and G3) grades [12-14]. Clinical staging in epithelial ovarian cancer (EOC) varies from stage I to IV. Stage I represents disease limited to one or both ovaries, stage II is associated with pelvic extension, stage III spreads into the abdominal cavity and stage IV presents distant metastases [reviewed in [11,15]].

A major problem with the diagnosis of a serous LMP tumor is that the absence of stromal invasion is the only feature that distinguishes them from invasive LG TOV tumors. The papilla of LMP serous tumors can be deeply invaginated in the stroma leaving doubt on the presence of invasion and can be dependant on the serial tissue section analyzed. In a subgroup of LMP tumors (10–15%), the presence of microinvasion is observed and consists of foci of invasive carcinoma in the ovarian stroma with a diameter smaller than 3 mm and covering a maximum surface area of 10 mm^2 ^[7,9,16-23]. Microinvasion does not appear to impact on patient prognosis [7,18,19,24,25]. LMP tumors may exhibit a specific architecture designated micropapillary serous carcinoma (MPSC) which is characterized by long and thin papilla (five times longer than larger) without hierarchical branching [26] [and reviewed in [19]]. Controversy persists as to the association of MPSC with a worse patient prognosis [26] [and reviewed in [19]]. A portion of LMP tumors are associated with peritoneal implants of epithelial or desmoplasic type. These implants are also characterized by their invasiveness. Invasive peritoneal implants are associated with a worse prognosis to LMP patients compared to the non-invasive implants [27,28].

From a clinical point of view, a diagnosis of serous BOV tumors does not interfere with survival. LMP tumors are also indolent and over 95% of LMP patients are still alive five years after their diagnosis. TOV tumors are the most lethal with only 30% of the patients surviving beyond five years. Patients diagnosed with a BOV or a low stage (SI-SII) LMP tumor undergo a conservative treatment based on the surgical removal of their tumor. The standard treatment regimen for advanced stage (SIII) LMP of high relapse risk and TOV patients is maximal cytoreduction followed by a platinium-taxane based chemotherapy [reviewed in [7,24]].

The relationship between serous BOV, LMP and LG TOV tumors at the molecular level remains unclear. Indeed, it has been suggested that the LMP class of tumors should be abolished and these tumors be subdivided into BOVs (LMP with typical architecture and/or non-invasive implants) and TOVs (LMP with MPSC architecture and/or invasive implants) [29], although this suggestion has not gained wide acceptance [19].

We previously identified candidate proteins differentially expressed between serous LMP and TOV tumors of various grades [30-32]. However, due to their rarity, LG tumors, were not well represented. Nonetheless, encouraging results were obtained for Ape1, Set, Trail, Ccne1 and Ran in their ability to discriminate LMP tumors. In the present study, our goal was to gain insight into the molecular relationship of BOVs, LMPs and LG TOVs. To this end, we constructed a tissue array composed of 27 BOVs, 78 LMPs and 23 TOVs of LG (Table 1) and evaluated protein expression of our previously identified candidates as well as three other EOC candidates (Ccnb1, Cdk1 and p21) identified in published microarray analyses which compared LMP and TOV tumors [33-36].

**Table 1:** Description of the serous tissue array composition

Patientes/samples characteristics	Tumor classification		
	
	BOV	LMP	LG
Number of tumors	27	78	23
			
Number of patients	25	56	18
			
Mean age ± S.D. (years)	48 ± 16	49 ± 14	52 ± 16
			
Disease Staging			
Stage I	-	25	1
Stage II	-	3	0
Stage III	-	27	15
Stage IV	-	-	1
no data available		1	1
			
Peritoneal Implants			
no	-	35	-
non-invasive	-	14	-
invasive	-	6	-
			
Presence of microinvasion			
no	-	49	-
yes	-	7	-

- not applicable

## Methods

### Patients and tissue specimens

Tumor samples were collected from patients who underwent surgery in the Division of Gynecologic Oncology at the Centre hospitalier de l'Université de Montréal (CHUM), the Centre hospitalier de l'Hôtel-Dieu de Québec (CHUQ) or the Centre hospitalier de l'Université de Sherbrooke (CHUS). The study was approved by the CHUM institutional ethics committee and written consent was obtained from patients prior to sample collection. Disease staging as defined by the Federation International of Gynecology and Obstetrics (FIGO) was determined by a gynecologist-oncologist. Histopathology and tumor grade were reviewed by an independent pathologist. Tissue selection criteria for this study were based on a serous histopathology from chemotherapy-naïve patients and all samples were collected between 1993–2005.

### Serous epithelial ovarian tumor tissue array

After a pathological revision of hematoxylin-eosin-stained slides, three representative cores (0.6 μm diameter) of each tissue sample, were arrayed on a recipient paraffin block. The tissue array was composed of 27 BOV, 78 LMPs and 23 LG tumors (Table 1). This tissue array was then sectioned, stained with hematoxylin-eosin and received another pathology review to confirm content. Tumor tissues from the three different institutions did not show statistically significant differences in protein staining (p > 0.10).

### Antibodies

For immunohistochemistry analysis, the following antibodies were used: anti-Ccne1 (sc-198) rabbit polyclonal antibody, anti-Ccnb1 (GNS1) mouse monoclonal antibody (sc-245), anti-Ran goat polyclonal antibody (sc-1156), anti-Cdc2 (Cdk1) (H-297, sc-747) rabbit polyclonal antibody, anti-p21 (C-19, sc-397) polyclonal antibody, anti-TRAIL goat polyclonal antibody (sc-6079), anti-I2PP2A (Set) goat polyclonal antibody (sc-5655) and anti-Ref-1 (Ape1) mouse monoclonal antibody (sc-17774) which were all purchased from Santa Cruz Biotechnology (Santa Cruz Biotechnology, CA, USA). Specificity of the antibodies was tested by Western blot.

### Immunohistochemistry

The tissue arrays, cut in 4 μm sections, were stained by an immunoperoxidase method as described elsewhere [30]. Tissue sections were heated to 60°C for 30 min, deparaffinized in toluene and rehydrated in an ethanol gradient. After 3% H_2_O_2 _treatment, slides were submerged in either boiling citrate buffer (0.01 M citric acid adjusted to pH 6.0) (Ape1, Set, Trail, Ccne1, Ran) or EDTA (Cdk1, Ccnb1, p21) for 15 min., blocked with a protein blocking serum-free reagent (DakoCytomation Inc., Mississauga, ON) and incubated with the antibody for 60 min at room temperature. Tissues were incubated with either a secondary biotinylated antibody (DakoCytomation Inc.) or a rabbit anti-goat biotin-conjugated antibody (1:300) (sc-2774, Santa Cruz Biotechnology) for 20 min followed by incubation with streptavidin-peroxidase complex (DakoCytomation Inc.) for 20 min at room temperature. Liquid diaminobenzidine was applied to visualize the reaction (DakoCytomation Inc.) and nuclei were counterstained with hematoxylin. Phosphate buffered saline was used instead of the primary antibody for negative control. Protein expression was scored according to the extent (as a percentage of total malignant cells) and intensity (value of 0 for absence, 1 for low, 2 for moderate, and 3 for high intensity) of staining based on visualization. These results were integrated in an algorithm currently used in the literature (% of cells with high intensity * 100) + ((% of cells with moderate intensity * 66.66) + (% of cells with low intensity * 33.33) [30-32]. Peripheral regions of the cores were not scored to eliminate edge effects. All slides were independently visualized by light microscopy at 20× magnification and scored in a blind study by two independent observers with an inter-rating of > 90%. When strong differences in scoring between the two observers occurred the core was re-evaluated to reach a consensus between the two observers.

### Statistical analysis

The association between immunohistochemistry staining score and tumor classification was analyzed by the Mann-Whitney U test. Comparisons of grades were performed between BOV, LMP and LG tumors. Statistical analysis was performed with SPSS software version 11 (SPSS Inc., Chicago, IL, USA) and statistical significance was set at p < 0.05.

## Results

**Figure 1:**
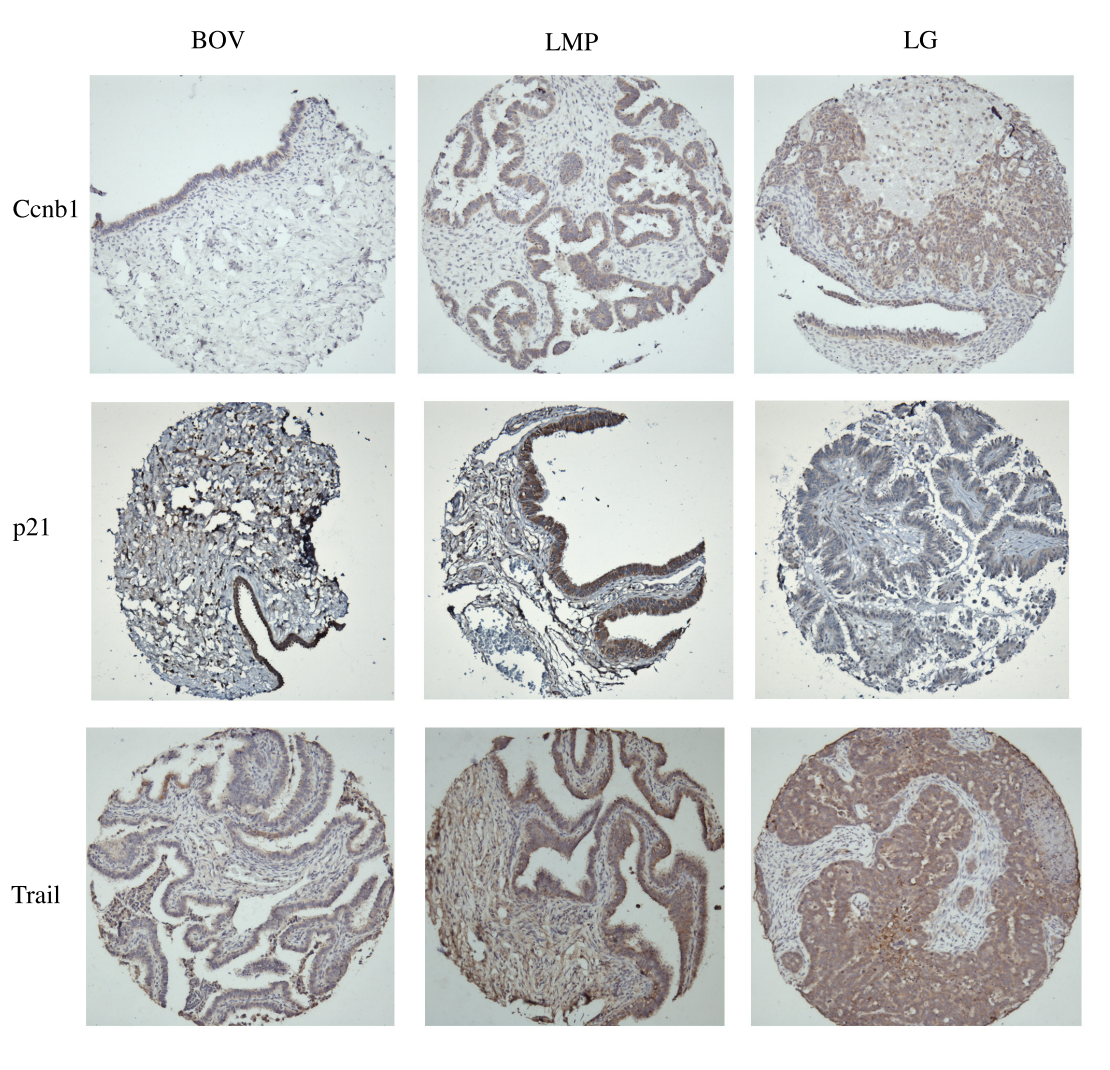
**Protein expression of Trail, p21 and Ccnb1 in serous epithelial benign, low malignant potential and LG ovarian tumors**. Representative images of immunoperoxidase-stained tissue cores are shown for each protein and tumor classes (20× magnification). Cytoplasmic and nuclear staining was observed for each of these proteins.

The mean score for expression was defined by the extent and intensity of immunohistochemistry staining for Ape1, Set, Trail, Ccne1 Ran, Ccnb1, p21 and Cdk1 (Figure 1). Statistically significant differences in protein expression were observed for Ccnb1 when BOV (low expression) were compared to LMP (high expression) tumors (p = 0.003) (Table 2). Comparing these two classes of tumors, protein expression of Trail presented a trend toward significance (p = 0.08) (Table 2). When BOV were compared to LG TOV tumors, Trail was significantly overexpressed in the malignant tumor (p = 0.01) (Table 2). LG TOV tumors presented statistically significant lower level of p21 compared either with BOV (p = 0.03) or LMP (p = 0.001) (Table 2). Finally, a trend toward significance of a higher expression of Ran in LG TOV compared to LMP tumors was observed (Table 2).

**Table 2:** Statistical analyses of candidate expression determined by immunohistochemistry and association to tumor class

Protein	Significance, Mann-Withney test evaluated with the mean score		
	
	BOV-LMP	BOV-LG	LMP-LG
Ape1	0.13	0.14	0.51
Hmgb2	0.98	0.78	0.59
Set	0.30	0.15	0.30
Trail	***0.08***	**0.01**	0.23
Ccnb1	**0.003**	0.25	0.20
Ccne1	0.20	0.64	0.16
Cdk1	0.19	0.75	0.15
p21	0.83	**0.03**	**0.001**
Ran	0.44	0.15	***0.10***

* bold mean that statistical significance is reached

* italic and bold mean that a trend toward statistical significance is reached

Some of the LMP tumors exhibited microinvasion (Table 1). Although the number of these cases is small (n = 8), we noted that LMP samples without microinvasion expressed higher level of Cdk1 (p = 0.05) compared to those showing microinvasion (data not shown). We also observed that microinvasive LMP tumors expressed higher levels of p21 (p = 0.01) and lower levels of Trail (p = 0.05) compared to LG TOV tumors (data not shown). Interestingly, we noted that expression of p21 was higher in LMP tumors associated with no (p = 0.02) or non-invasive (p = 0.01) implants compared to the LMP with invasive implants (data not shown).

## Discussion

Only a few studies focus have focused on the molecular characterizing serous BOV, LMP and LG TOV tumors, but the biological markers distinguishing between these three classes of tumors remains to be defined. In our previous studies, protein expression of selected candidates showed statistically significant differences between serous LMP and LG TOVs, although the invasive tumors, due to their rarity, were under-represented. Based on these results and the necessity to better understand the relationship between BOV, LMP and LG TOV epithelial ovarian serous tumors, we constructed a tissue microarray focusing on BOV, LMP and LG TOV tumors.

Among all candidate proteins tested (Ape1, Set, Trail, Ccnb1, Ccne1, Cdk1, p21 and Ran), the most interesting results were obtained with p21 (Cdkn1a, Waf1, Cip1). Expression of this protein is highly regulated by p53 and acts like its effector [37,38]. P21 plays a role in cell cycle regulation by inhibiting the cyclin dependant kinase (Cdk) [39,40] [and reviewed in [41,42]]. Low expression of p21 was observed in many types of cancer including those of the ovary [34,35,43-47], colon [48], lung [49,50], head and neck [51], bladder [52], gastric [53], endometrium [54] and oral cancer [55]. Decreased p21 expression was shown to be inversely associated with the index of genomic instability in tumor associated with the worse prognosis to the patient [56,57].

In the present study, statistically significant higher expression of p21 was observed in BOVs, LMPs or the subgroup of microinvasive LMPs when compared to LG TOVs. These results are consistent with those found in the literature showing that decreased p21 expression was associated with aggressiveness of the tumors and/or poor patient prognosis defined by response to chemotherapy, disease free interval and/or overall survival [34,35,43-46]. However, we were not able to reproduce previous results showing a significant underexpression of p21 in BOV compared to LMP tumors [58].

Interestingly, we observed a lower expression of p21 in the subgroup of LMPs with invasive peritoneal implants that are known to confer a worse prognosis in term of progression and overall survival to the LMP patient [27,28]. To our knowledge, this is the first demonstration of differential expression of a protein within LMP tumors. These results support the role of p21 in the aggressiveness and/or invasiveness of ovarian tumors. However, further studies would be required in order to increase the number of these rare samples of LMP presenting invasive implants. Another differentially expressed protein is Ccnb1. LMP tumors expressed higher level of Ccnb1 compared to BOV. This result correlates with those presenting an overexpression of *CCNB1 *in highly malignant or poorly differentiated ovarian tumors when monitored by cDNA microarray analysis [33,35,36,59]. Overexpression of Ccnb1 was also observed in lung [60,61] and gastrointestinal [62] cancer.

In our previous studies, we showed statistically significant differences in the expression of Ape1, Set, Trail, Ccne1 and Ran between serous LMPs and LG TOVs [30-32] based on a limited number of samples for the LG tumors. Within the extended series presented here, only Ran continued to show a trend toward significance within these LG tumors. These results demonstrate the importance of validating candidate markers on the largest possible number of samples in order to ensure their ability to discriminate between two groups of tumors. Our previous results, based on a large set of samples, indicate that Ape1, Set, Trail, Ccne1 and Ran can discriminate serous LMPs from high grade TOVs [30-32]. The dualistic model of EOC tumor development suggests that a large portion of high grade EOC tumors (G2 and G3) arise from the ovarian epithelium cells by as yet not clear mechanism implicating *TP53*, *BRCA1 *and/or *BRCA2 *mutations [63], whereas the LG TOVs (grade 1) arise from a sequence of unknown molecular events beginning with the development of BOV, transitioning through to LMP and MPSC before ending with a LG TOV tumor [63,64]. This continuum is supported by the fact that serous BOVs and LMPs share characteristics of LG TOVs as opposed to high grade tumors. [reviewed in [19,65,66]]. While it would be tempting to speculate that our combined results support the dualistic model of tumor progression, we cannot exclude the possibility that this represents a model where significant differences could only be seen in higher grade tumors following tumor progression [13,63]. In line with this notion, we observe that the protein level of Trail was significantly lower in BOVs when compared to LG tumors and tends toward significance when compared to LMPs. These results combined to our previous study [32] could suggest an incremental increase of Trail expression from BOV to LMP, LG, G2 and G3 tumors. These results are in line with those seen in colon cancer where Trail expression is lower in adenoma when compared to adenocarcinoma [67].

## Conclusion

In conclusion, we provide one of the few studies that simultaneously evaluated differential expression of selected proteins on serous BOV, LMP and LG EOC tumors. We showed that protein expression of Ccnb1 and Trail can distinguish BOV from LMP and LG TOVs respectively. We highlight the differential expression of p21 between the LMP without or with non-invasive implants compared to the more aggressive LMPs presenting invasive implants and suggest that this result be validated in the future on a larger set of LMPs with invasive implants. The p21 results may ultimately be useful to identify the rare poor outcome patient with LMPs and exploited for the management of these patients.

## Abbreviations

BOV: Benign ovarian tumor; LMP: Low malignant potential tumor; TOV: Malignant ovarian tumor; G: Tumor grade; EOC: Epithelial ovarian cancer; MPSC: Micropapilary serous carcinoma.

## Competing interests

The authors declare that they have no competing interests.

## Authors' contributions

VO performed staining and statistical analyses as well as writing and editing of the manuscript according to all authors revisions. THL, KN and VB performed immunohistochemistry and staining analysis. VO and THL performed the selection of patient samples and revision of patient clinical data. JM built the tissue microarray. CL performed the pathological review of the slides and tissue microarray. CR, DB, PNT, DMP and AMMM contributed to the conception and design of the study as well as analysis and interpretation of the data. All authors revised the manuscript and gave final verbal approval.

## Pre-publication history

The pre-publication history for this paper can be accessed here:


